# A Challenging Case of Heart Displacement by a Large Mediastinal Germ Cell Tumor

**DOI:** 10.7759/cureus.41762

**Published:** 2023-07-12

**Authors:** Mohammed E Almalki, Mansour M Almalki, Mohammed A AlHarbi, Abdulkareem Nmnkany, Mona H Tayib, Fatma Aboul Enein, Saleh M Khouj

**Affiliations:** 1 Department of Medicine, College of Medicine, Umm Al-Qura University, Makkah, SAU; 2 Department of Cardiology, Alexandria University, Alexandria, EGY; 3 Department of Cardiology, King Abdullah Medical City, Makkah, SAU; 4 Department of Interventional Cardiology and Structural Heart Disease, King Abdullah Medical City, Makkah, SAU

**Keywords:** mediastinal shift, chemotherapy, cardiac tamponade, non-seminomatous germ cell tumor, mediastinal germ cell tumors

## Abstract

Mediastinal germ cell tumors (GCTs) are rare and aggressive cancers originating from the germ cells in the mediastinum. Early detection and treatment are vital due to their high potential for metastasis and recurrence. We present a case of a 28-year-old man who exhibited a cough and shortness of breath. Laboratory tests revealed elevated tumor markers, alpha-fetoprotein, and beta-human chorionic gonadotropin. Imaging studies displayed a large mediastinal mass, causing the right displacement of the mediastinum and cardiac tamponade. The biopsy confirmed a non-seminomatous GCT, specifically a yolk sac tumor. The patient experienced pericardial effusion and cardiac tamponade after receiving two cycles of etoposide and cisplatin chemotherapy. To relieve the tamponade, an emergency pericardiocentesis was performed malignant GCTs necessitate prompt diagnosis and treatment and utilizing multimodal therapy such as chemotherapy to achieve tumor control. Due to the high risk of metastasis, vigilant surveillance for recurrence is essential, emphasizing the need for specific criteria for accurate early detection.

## Introduction

Testicular neoplasms are the most common solid organ malignancies in males aged between 15 and 35 years [1]. Extragonadal germ cell tumors (GCTs) are relatively uncommon types that account for 1% to 5% of all GCTs. They can be found in various anatomic locations but most frequently in the mediastinum and sacrococcygeal areas [2]. Primary mediastinal GCTs originate from primitive germ cells that have migrated abnormally along the urogenital ridge during embryonic development [3].

Mediastinal non-seminomatous GCTs account for two-thirds of mediastinal GCTs [4]. The prognosis of this type is poorer than that of its gonadal and retroperitoneal analogs [5]. The survival rate for the non-seminomatous histological type after five years was reported to be between 40% and 45% [5]. The course of treatment has changed over the past 30 years and depends on the histology and complications present at the initial presentation. According to the literature, cisplatin-based chemotherapy and consolidative local therapy, such as surgery or radiotherapy, are the recommended treatments [6-8].

We report a case of a mediastinal non-seminomatous GCT metastasized to the liver, lung, vertebrae, and brain after two cycles of etoposide and cisplatin chemotherapy (EP), which was then lost to follow-up. The patient had presented with acute cord compression and underwent laminectomy with fixation. A transthoracic echocardiogram revealed a moderate-to-large accumulation of fluid around the heart along with a mass, leading to a diagnosis of pericardial effusion with tamponade. Before performing pericardiocentesis, a computed tomography (CT) scan was performed, which demonstrated a right-sided shift of the mediastinum.

## Case presentation

A 28-year-old male Arabic patient was transferred in November 2021 from a secondary hospital to a tertiary center with a chief complaint of cough, shortness of breath, and a mediastinal mass on the CT scan for further evaluation. Ultrasound-guided lung biopsy taken on the day of the presentation showed a picture favoring a yolk sac tumor, confirming the diagnosis of a non-seminomatous GCT of the mediastinum. He first presented to the oncology clinic with the same complaint and lower limb weakness. On further questioning, he denied a family history of malignancies but revealed that his father was diabetic and hypertensive. The patient smoked 10 to 20 cigarettes a day but stopped smoking six months prior. On physical examination, the patient was mentally alert with a Glasgow Coma Scale of 15/15. He was cachectic and in a wheelchair. His blood pressure was 137/63 mmHg, pulse 120 beats per minute, and respiratory rate 20 breaths per minute. Breath sounds were reduced over the left lung; no additional sounds were heard, and there was chest wall tenderness. Abdominal examination was unremarkable. Motor examination revealed lower limb power of 1/5 bilaterally and bilaterally intact sensation. The patient was admitted to begin treatment and undergo further investigations. Laboratory results are summarized in Tables 1-2.

**Table 1:** Patient’s serum biomarker levels.

Test	Result	Normal range
Beta-human chorionic gonadotropin	15.36 IU/L	>2 IU/L
Alpha-fetoprotein	69,221.70 µg / L	0-40 µg / L
Lactate dehydrogenase	1,058 IU/L	87-241 IU/L

**Table 2:** Complete blood chemistry and hematological profile results. ALT, alanine aminotransferase; AST, aspartate transaminase; RDW-CV, red cell distribution width-coefficient of variation

Test	Result	Normal range
Blood urea nitrogen	7.09 mmol/L	2.86-8.57 mmol/L
Creatinine, serum	54.81 µmol/L	65.42-119.34 µmol/L
Uric acid, serum	1.698 mmol/L	1.234 -2.543 mmol/L
Potassium, serum	5.6 mmol/L	3.5-5.1 mmol/L
Sodium, serum	134 mmol/L	136-145 mmol/L
Magnesium, serum	0.85 mmol/L	0.66-1.07 mmol/L
Phosphorus, serum	1.29 mmol/L	0.8-1.45 mmol/L
Calcium, serum (total)	2.6 mmol/L	2.2-2.58 mmol/L
Calcium (corrected)	2.92 mmol/L	2.12-2.52 mmol/L
Albumin, serum	0.024 g/L	0.034-0.05 g/L
ALT	25 U/L	12-78 U/L
AST	31.0 U/L	8-48 U/L
Alkaline phosphatase	92 U/L	45-115 U/L
Bilirubin (total)	13.17 µmol/L	<20.52 µmol/L
Bilirubin (direct)	6.84 mmol/L	0-5.13 mmol /L
Lipase	60 U/L	73-393 U/L
Hemoglobin	121 g/L	135-175 g/L
Hematocrit	41.1%	37%-53%
Platelet count	398 L^-1^	150 × 10^9^ to 400 × 10^9^ L^-1^
RDW-CV	16.2%	11.8%-15.6%
White blood cells	7.14 L^-1^	3.9 × 10^9^ to 11 × 10^9^ L^-1^
Neutrophils	4.75 L^-1^	2.5 × 10^9^ to 7 × 10^9^ L^-1^
Lymphocyte	19%	20%-45%
Monocyte	12.3%	2%-8%
Eosinophils	1.4%	0%-6%
Basophils	0.7%	0%-1%

**Figure 1:**
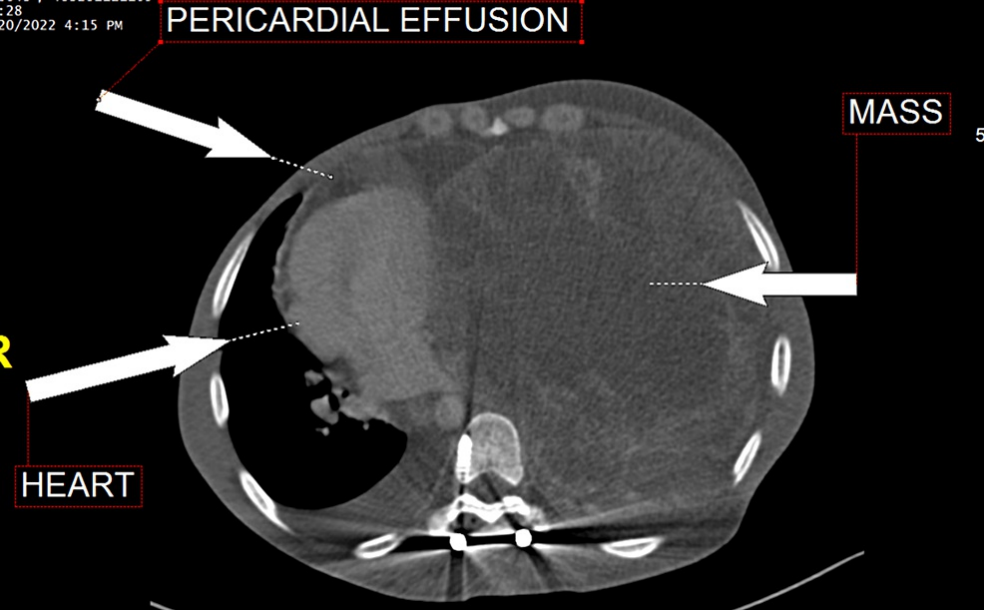
Contrast-enhanced chest CT revealing a large lobulated heterogeneous mediastinal mass. CT, computed tomography

Initial imaging included a chest CT with contrast (Figure 1), which showed a large lobulated heterogeneous mediastinal mass, measuring approximately 15 cm × 18.4 cm × 22.6 cm and demonstrating a significant interval increase in size. The lesion filled most of the left hemithorax, causing a significant mediastinal shift to the right side. The lesion exhibited inseparability from the costal and mediastinal pleurae with mild left and minimal right pleural effusions. There were no findings of underlying major pulmonary embolism. The right basal segment showed an 8 mm medial pulmonary nodule, suggesting the possibility of metastasis.

**Figure 2:**
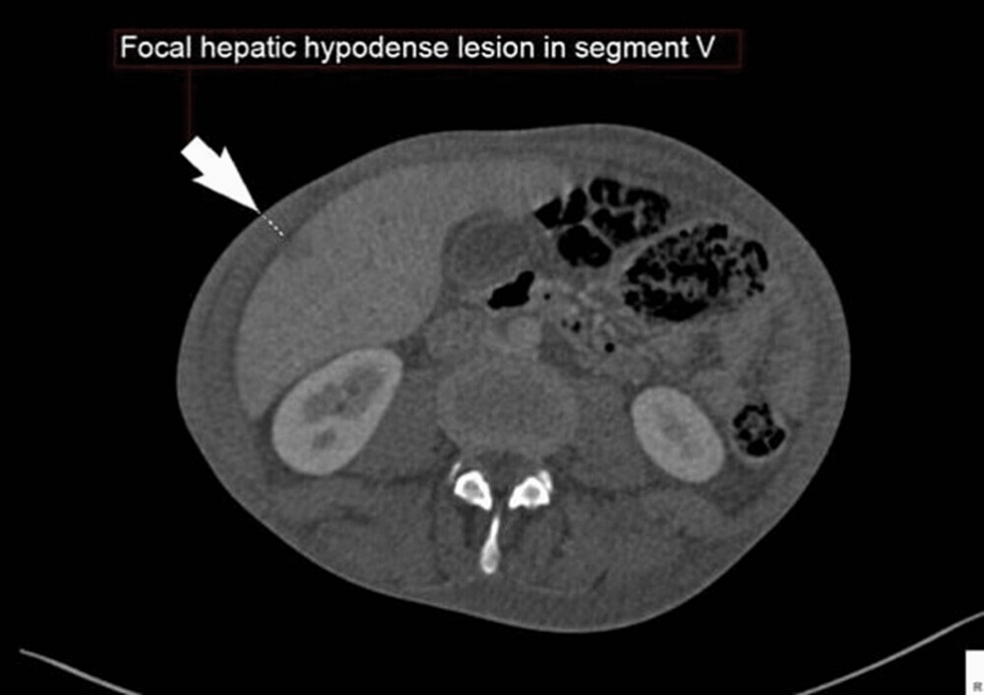
In this axial view of an upper abdominal CT scan, a metastatic lesion in segment V of the liver is clearly visible (arrow). CT, computed tomography

The patient was started on chemotherapy. The first cycle was composed of two medications: cisplatin 22.96 mg in 500 mL normal saline infusion plus etoposide (100 mg/5 mL) 114.8 mg intravenous infusion in normal saline every 24 hours for five days, in addition to heparin sodium infusion and filgrastim (G-CSF) 300 µg/mL solution for five days. The patient was classified as a high-risk chemotherapy protocol and started on preventive treatment for antitumor lysis syndrome. He was also receiving supportive medication to minimize the side effects of chemotherapy, such as omeprazole, netupitant, palonosetron, dexamethasone sodium phosphate, metoclopramide, and opioid analgesics such as morphine sulfate and tramadol. After chemotherapy, investigations were carried out to evaluate the response to treatment. Chest, abdominal, and pelvic CT with contrast was done and revealed a complete collapse of the left lung with a mild increase in the pericardial effusion and mediastinal shift, a hypodense hepatic lesion in segment V (Figure 2).

 

Transthoracic echocardiography was performed with limited views showing a moderate-to-large pericardial effusion (Video 1).

**Video 1:** Moderate-to-large circumferential pericardial effusion with right atrial and ventricular diastolic collapse detected by transthoracic echocardiography.

On February 2022, the patient came to the emergency room with worsening symptoms of cough, shortness of breath, tachypnoea, and palpitations.

The patient was desaturated but conscious, coherent, and oriented to time and place on examination. He was placed on bilevel positive airway pressure (BIPAP) and had a chest X-ray. Urgent cardiology consultation and echocardiogram revealed a moderate-to-large circumferential pericardial effusion with right atrial and ventricular diastolic collapse, consistent with cardiac tamponade. A large heterogeneous mass was also visualized to the left of the heart. The patient was moved to the cardiac catheterization laboratory for urgent pericardiocentesis, where 400 cc of reddish fluid was removed and a pericardial drain was connected. Cytology of the pericardial fluid reveals a hemorrhagic effusion with high white blood cell count and abundant polymorphonuclear leukocytes, suggestive of an inflammatory process. The patient was then transferred to the intensive care unit. He was sedated with fentanyl at a rate of 100 µg/hour, and a vasopressor (levophed) was initiated at a rate of 0.35 µg/kg per minute. The patient's condition did not improve, and levophed was increased to 4 µg/kg per minute with 5% dextrose at an infusion rate of 80 mL/hour. As there was no response following the administration of epinephrine, a 500 mL infusion of Ringer's lactate was administered. The patient received cardiopulmonary resuscitation twice for 30 minutes but died after one day in the intensive care unit. Consent for the publication of this case report was obtained from the patient's family.

## Discussion

Extragonadal GCTs are divided into two main types: seminoma and non-seminoma. Seminoma GCTs have a better prognosis and are radiosensitive [9,10] compared with the vast majority of the cases that fall within the category of non-seminomatous GCTs. The latter are subdivided according to the type of cell into teratocarcinoma, embryonal carcinoma, choriocarcinoma, mixed tumors, and yolk sac carcinoma [11]. Non-seminomatous GCTs are less sensitive to radiotherapy but sensitive to chemotherapy, unlike seminomas, which are sensitive to both [9,10].

Extragonadal GCT is a form of GCT where the sperm go outside the reproductive organ and grow [12]. The best way to rule in, or rule out, the presumptive diagnosis would be via assessment of the patient’s clinical presentation. This includes taking a detailed history and carrying out a physical examination and different types of tests, such as serum markers, studies, and biopsies to identify the presence of tumors, assess their potential for metastasis, and determine the risk of metastasis.

Primary extragonadal GCTs are rare, but when they do happen, their most common target sites are the retroperitoneal area or, as in our case, the mediastinum. They have the same components as gonadal tumors histologically, but they may differ in their clinical and biological features [13,14].

Patients with mediastinal non-seminomatous GCT typically exhibit symptoms such as chest pain, cough, hemoptysis, or weight loss at the time of diagnosis. Pericardial tamponade or superior vena cava syndrome may also be present [15,16]. In our case, the patient came complaining of a cough and shortness of breath.

Serum markers including alpha-fetoprotein, beta-human chorionic gonadotropin, and lactate dehydrogenase can all help establish a diagnosis, while the definitive diagnosis is being pursued via biopsy [16,17]. In the case of mediastinal primary non-seminomatous tumors, we can find alpha-fetoprotein marker levels higher than 10,000 ng/mL, human chronic gonadotropin higher than 50,000 IU/L, and lactate dehydrogenase 10 times higher than the upper limit of normal [18]. In our case, the level of alpha-fetoprotein was 69,221.1 µg/L, beta-human chronic gonadotropin was 15.36 IU/L, and lactate dehydrogenase was 1,058 IU/L. Ultrasound-guided biopsy was performed, and it confirmed the diagnosis of non-seminomatous GCT of the mediastinum favoring a picture of a yolk sac tumor.

In the context of our case, the significance of utilizing advanced imaging modalities, such as CT scans, for cancer patients cannot be overstated, particularly when performing pericardiocentesis. These modalities play a crucial role in enhancing the safety and efficacy of the procedure. By providing visual guidance, they help mitigate the risks associated with blindly inserting a needle, which could potentially result in internal bleeding or damage to structures adjacent to the pericardium. In our specific scenario, the presence of a sizable mediastinal mass necessitated a more cautious approach. The abnormal mass had caused a complete rightward shift of the heart, thereby altering the conventional orientation of anatomical structures. Consequently, relying solely on traditional techniques would have carried a higher risk of complications. However, through careful assessment of the patient's imaging studies and ultrasound guidance, we were able to devise a modified procedure. By incorporating ultrasound guidance, we strategically directed the needle toward the right side rather than following the customary trajectory. This tailored approach allowed us to safely and successfully drain the pericardial fluid without encountering any adverse events. Our experience reaffirmed the critical importance of thoroughly reviewing all available imaging modalities before proceeding with invasive procedures.

## Conclusions

In conclusion, prompt diagnosis and treatment are crucial in managing malignant GCTs, and multimodal therapy, including chemotherapy, is often necessary to achieve tumor control. Due to the high risk of metastasis, vigilant surveillance for recurrence is essential, emphasizing the importance of specific criteria for accurate early detection. Advanced imaging modalities, such as CT scans, play a vital role in ensuring the safety and effectiveness of procedures like pericardiocentesis in cancer patients. These imaging techniques provide precise visualization of anatomical variations caused by mediastinal masses, enabling healthcare professionals to make informed decisions and adapt their approach accordingly. Our successful case underscores the value of incorporating ultrasound guidance and thoroughly evaluating imaging modalities to mitigate potential complications and enhance patient outcomes.
